# Enhancing accuracy in brain stroke detection: Multi-layer perceptron with Adadelta, RMSProp and AdaMax optimizers

**DOI:** 10.3389/fbioe.2023.1257591

**Published:** 2023-09-25

**Authors:** Mudita Uppal, Deepali Gupta, Sapna Juneja, Thippa Reddy Gadekallu, Ibrahim El Bayoumy, Jamil Hussain, Seung Won Lee

**Affiliations:** ^1^ Chitkara University Institute of Engineering and Technology, Chitkara University, Punjab, India; ^2^ KIET Group of Institutions, Ghaziabad, India; ^3^ Zhongda Group, Jiaxing, Zhejiang, China; ^4^ Department of Electrical and Computer Engineering, Lebanese American University, Byblos, Lebanon; ^5^ School of Information Technology and Engineering, Vellore Institute of Technology, Vellore, India; ^6^ College of Information Science and Engineering, Jiaxing University, Jiaxing, China; ^7^ Division of Research and Development, Lovely Professional University, Phagwara, India; ^8^ Public Health and Community Medicine, Tanta Faculty of Medicine, Tanta, Egypt; ^9^ Department of Data Science, Sejong University, Seoul, Republic of Korea; ^10^ Sungkyunkwan University School of Medicine, Suwon, Republic of Korea

**Keywords:** heart stroke, multi layer perceptron, AdaMax, RMSProp, Adadelta, deep learning

## Abstract

The human brain is an extremely intricate and fascinating organ that is made up of the cerebrum, cerebellum, and brainstem and is protected by the skull. Brain stroke is recognized as a potentially fatal condition brought on by an unfavorable obstruction in the arteries supplying the brain. The severity of brain stroke may be reduced or controlled with its early prognosis to lessen the mortality rate and lead to good health. This paper proposed a technique to predict brain strokes with high accuracy. The model was constructed using data related to brain strokes. The aim of this work is to use Multi Layer Perceptron (MLP) as a classification technique for stroke data and used multi-optimizers that include Adaptive moment estimation with Maximum (AdaMax), Root Mean Squared Propagation (RMSProp) and Adaptive learning rate method (Adadelta). The experiment shows RMSProp optimizer is best with a data training accuracy of 95.8% and a value for data testing accuracy of 94.9%. The novelty of work is to incorporate multiple optimizers alongside the MLP classifier which offers a comprehensive approach to stroke prediction, providing a more robust and accurate solution. The obtained results underscore the effectiveness of the proposed methodology in enhancing the accuracy of brain stroke detection, thereby paving the way for potential advancements in medical diagnosis and treatment.

## 1 Introduction

Brain stroke is the most serious and fatal illness that affects people. The rising incidence of brain stroke that is linked to an increased death rate had a significant concern to healthcare systems around the world. Everyone knows that the human brain is a complex part of the body. The brain’s intellectual center, movement maker, sensation interpreter and behavior controller weighs approximately three pounds. This region of the brain regulates important human functions like emotion, cognition, respiration, memory, vision, touch, motor skills, hunger, and temperature. The brain, which is contained in a bone shell and maintained clean by a lubricating fluid, is the origin of all the traits that make us human (Sirsat ManishaSanjay et al., 2020). When the blood supply to the brain is restricted and tissues of the brain are deprived of nutrients and oxygen, then a brain stroke happens. This causes cells of the brain to start dying within a minute. Every day, there are more and more people experiencing a stroke. Males are more likely than females to get a brain attack, especially as they get older. On the other hand, 8% of kids with sickle cell illness experience stroke. Every year, 15 million people experience a stroke worldwide (Singh et al., 2020). Many families and communities are impacted by the fact that 5 million of these patients are passed away and another 5 million will become permanently disabled. So, a prediction model is required to help clinicians to identify stroke by putting patient information into a processing system in order to lessen the mortality of patients having a brain stroke. It can devastate the healthcare system globally, but early diagnosis of disorders can help reduce the risk (Gaidhani et al., 2019; Bandi et al., 2020). When an artery to the brain is jammed, the affected area’s brain cells die from a lack of oxygen. It frequently happens in areas with poor and intermediate incomes (Bonita, 1992). Strokes either can be hemorrhagic or ischemic that refers to a lack of bleeding or blood flow respectively. Both conditions block certain functions in their respective tracks. The incapability to move around or feel either side of the body, difficulty in speaking or understanding something, loss of vision and dizziness are possible symptoms of a brain stroke (Lee et al., 2021). A Transient Ischemic Attack (TIA) is a kind of brain stroke where symptoms last only less than 1 or 2 h (Weih et al., 1999).

**FIGURE 1:**
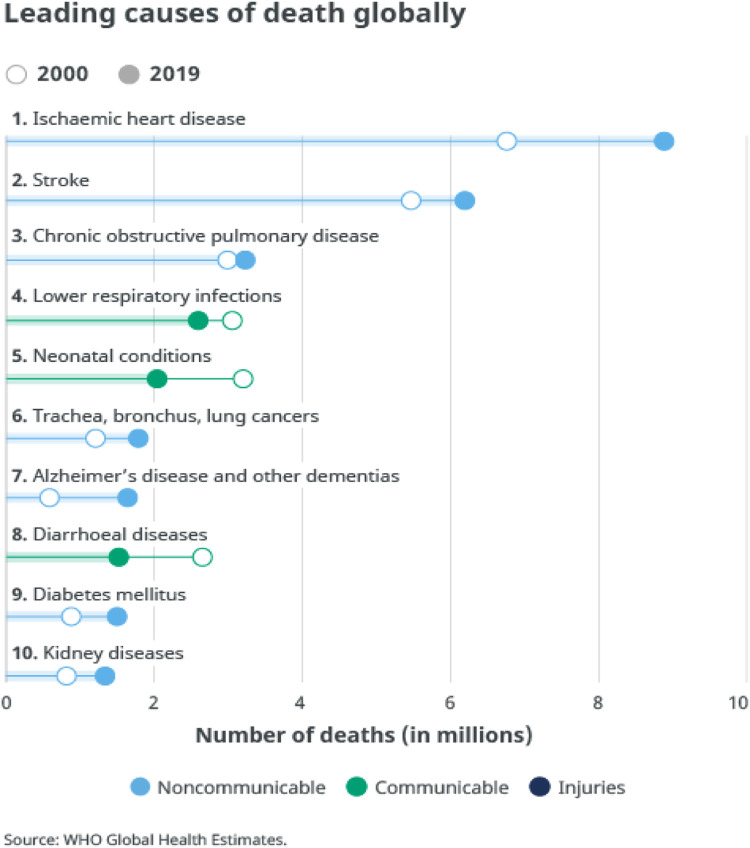
Leading causes of death globally, recorded in 2019 (World Health Organization, 2020).

Figure 1 illustrates the most recent global health estimates from 2000 to 2016, showcasing the leading causes of mortality and disability. It highlights ischemic heart disease and stroke as the top two contributors to these statistics worldwide (Johnson et al., 2016). Stroke, as per the American Heart Association, is a significant health concern due to its high fatality rate (Benjamin et al., 2018). Moreover, the expenses associated with stroke-related hospitalizations are on the rise and there is demand of advanced technologies (Di Carlo, 2009; Fermé et al., 2020). Stroke ranks as the 2nd leading cause of death, accounting for around 11% of all deaths. Over the years, deep learning (DL) has experienced rapid growth and advancement, finding diverse applications across various healthcare systems. Detecting stroke at an early stage is a critical aspect of effective treatment, and deep learning holds substantial potential in this domain.

Stroke is a highly significant medical and social issue that necessitates prompt attention. One crucial aspect of stroke management is accurately predicting the occurrence of strokes based on available datasets. A key challenge lies in achieving improved performance and accuracy in stroke prediction compared to traditional methods employed by radiologists and doctors. The implementation of deep learning models can aid in addressing this problem by enabling timely treatment for patients (Gupta, 2019). Radiologists often rely on computer-aided diagnosis (CAD) systems to enhance the accuracy of their predictions. CAD systems assist in improving the efficiency and accuracy of stroke predictions made by radiologists (Tang et al., 2011).

**FIGURE 2:**
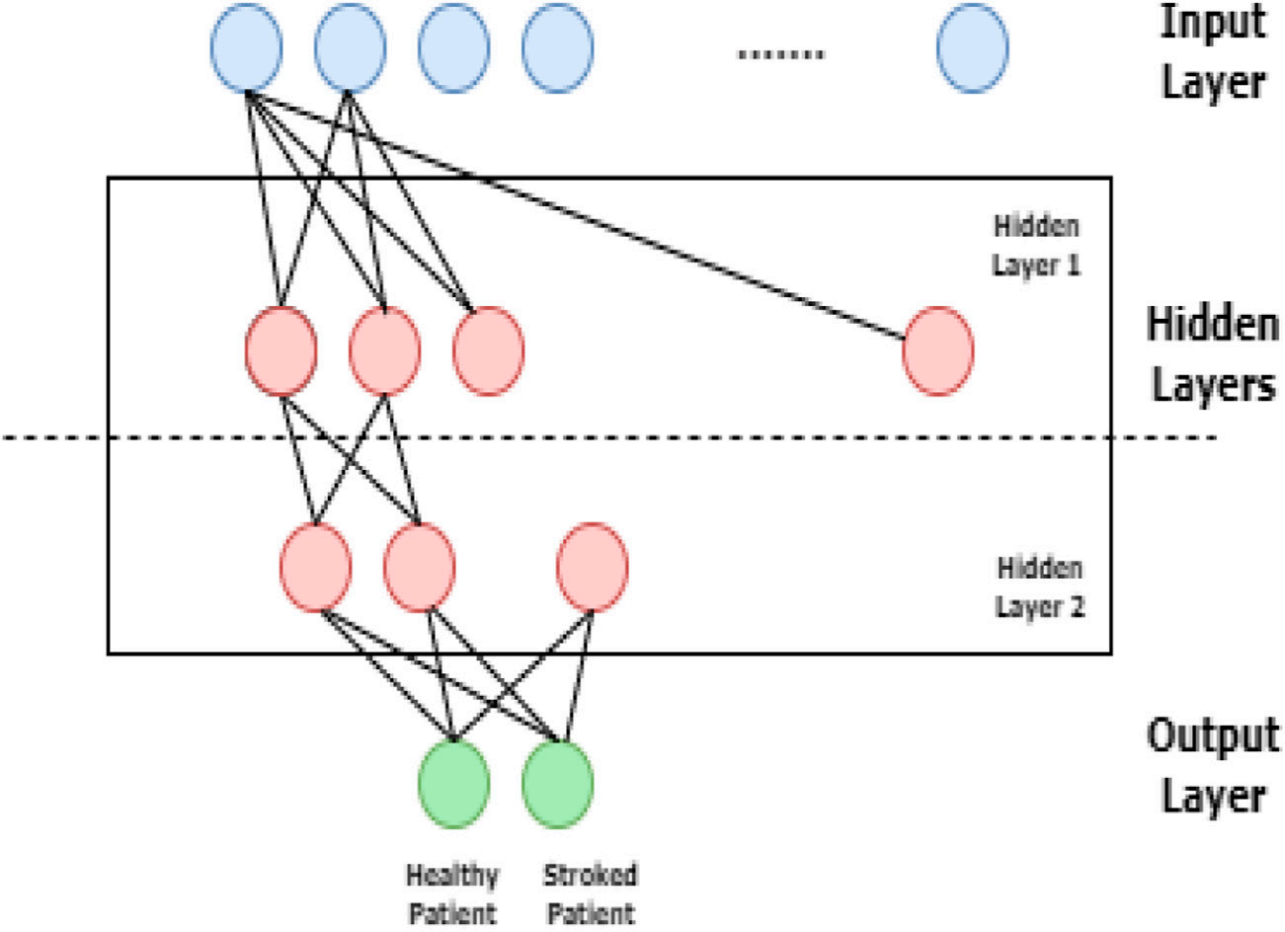
Logical representation of Multi-Layer Perceptron.

Deep learning can forecast the beginning of brain stroke because of advances in medical field (Chin et al., 2017a). The techniques used in deep learning provide proper and precise analysis and produce accurate predictions. The most popular neural network model when employing the back-propagation training process is the multilayer perceptron. The architecture of MLP networks is important consideration because too many connections may over fit the training data and too few connections may stop network from decoding problem of inadequate customizable parameters (Bonita, 1992; Shin et al., 2022). MLPs are feed forward neural networks that consist of single input layer, one or more hidden layers and single output layer. The neurons present in input layer obtain the input data, which is then passed through the hidden layers to the output layer as shown in below Figure 2.

Each neuron in the MLP has an associated weight vector that specifies the power of connection between different neurons in the adjacent layers. The weights are accommodated to lessen the error between the predicted and the actual outputs of the network using a supervised learning algorithm during training. The activation function used in output layer of MLP determines nature of the problem being solved. For example, if the problem is a binary classification task, a sigmoid or a softmax function can be used. In the case of regression task, tanh or linear function is more suitable. Path tracking technique is an example of MLP which shows how the tasks are controlled. MLP is used in different applications like speech recognition, image recognition or natural language processing (Lins and Bernarda Ludermir, 2005). Bibliometric analysis is used to analyze and predict the trends in a specific field that gives a visual representation of information. In the medical field, this analysis helps in gaining deep insights in different areas of research yet its application in the brain stroke domain is limited. With an increase in the number of publications, there is a need to update research data through bibliometric analysis that is specific to the brain stroke domain (Kokol et al., 2021).

Brain stroke is a medical emergency that needs a diagnosis that can bring a difference between death and life of a person which can either lead to full recovery or permanent disability. By increasing the accuracy using multiple optimizers, medical technology is contributing to the advancement of brain stroke detection that will save life and enhance the life of patients. So, the motivation behind this work is to make a difference in the life of a person affected by a brain stroke. This research work emphasizes stroke diagnosis and prevention which has a significant role in the medical field. It is a challenging task to diagnose strokes in patients that are residing in remote areas where there is a limitation of healthcare facilities. Identification of stroke is a time-consuming process so this paper develops a model that helps in solving these issues which are associated with brain stroke disease and improves the decision-making process. The amalgamation of medical work with ML techniques in the field of stroke detection and prevention leads to great heights where ML model help specialists to make good decision and have reliable support. Through this work, the aim is to achieve the following objectives:

• To conduct a thorough literature review to analyze existing research in the detection of brain stroke.
• To develop a novel method for improving the accuracy of brain stroke detection using Multi-Layer Perceptron using Adadelta, RMSProp and AdaMax optimizers.
• To evaluate the performance of Adadelta, RMSProp and AdaMax optimizers individually in improving the accuracy of brain stroke detection.
• To compare the accuracy levels of each optimizer (Adadelta, RMSProp, and AdaMax) in the context of brain stroke detection, aiming to identify the optimizer that yields the highest accuracy and suitability for stroke detection.

The rest of the paper is structured as follows: Section 2 presents the related work conducted in the field of brain stroke detection. In Section 3, the materials and methods employed in this work are explained that include details about the dataset, introduction to deep learning and its optimizers and the proposed methodology. The experimentation and analysis of the results on different splits are presented in Section 4. Section 5 concludes the paper and discusses future work for future research and development.

## 2 Related work

Deep learning is a potential tool for early sickness prediction and numerous research has been done to forecast diseases including cancer, stroke illnesses, skin conditions and many more. There is very less research on prediction of brain stroke.

Five ML algorithms are applied to the dataset provided by Cardiovascular Health Study (CHS) to forecast the strokes (Singh et al., 2020). The authors have employed a combination of various ML algorithms and techniques to predict stroke and identify stroke-related symptoms. The use of a Decision Tree with the C4.5 algorithm, ANN, PCA and SVM can potentially provide a more accurate prediction of stroke by utilizing different approaches to handle different aspects of the dataset. The use of social media content to predict stroke is an interesting approach (Pradeepa et al., 2020). However, the use of NLP to extract text from posts can increase overall execution time of model that may not be desirable in real-world applications where predictions need to be made in a timely manner. It is also worth noting that social media posts may not always provide reliable information, and there may be privacy concerns associated with accessing and analyzing user-generated content. In (Bandi et al., 2020), authors proposed an adapted random forest algorithm for stroke prediction and evaluated the risk levels related to stroke. This approach performed better as compared to other algorithms, as it is restricted to a less number of stroke types only. According to (Nwosu et al., 2019), the proposed model was trained using random forests, decision trees and MLP to predict strokes. Decision Tree’s accuracy was calculated to be 74.31%, Multi-layer Perceptron’s accuracy to be 75.02% and Random Forest’s accuracy was 74.53%. The research work (Alotaibi, 2019) predicted cardiac attacks using an ML algorithm. The authors built a model using a variety of ML approaches like Decision Tree, SVM and Naive Bayes and their results were compared. The proposed method yielded an accuracy of just 60%. In (Almadani et al., 2018), the authors forecasted the possibility of brain stroke using Jrip, C4.5 and MLP classification techniques. The proposed model was approximately 95% accurate but training and testing times are more as the authors used various complex algorithms. Three distinct algorithms, namely, Neural Networks, Decision Trees and Naive Bayes are suggested by (Kansadub et al., 2015) for use in predicting the occurrence of stroke. This paper found that DT has maximum accuracy (around 75%). To improve efficiency and quality of life, suggested approach uses an ML algorithm to continuously check the health of IoT devices. The researchers looked into the current state of AI technology in the healthcare industry and evaluated its future potential. Their goal was to use bibliometric analysis to evaluate the evolution of the relationship between AI and digital health approaches. Additionally, they took into account the moral considerations and ethical AI practices built into this field’s technological breakthroughs (Soni et al., 2023a; Soni et al., 2023b). To predict stroke, the authors used the Cardiovascular Health Study (CHS) dataset and formed a novel automated feature selection technique (Khosla et al., 2010). They integrated this technique with SVM algorithm to achieve better efficiency and created many vectors which have a tendency to make the model perform worse. (Shanthi et al., 2009). proposed to use ANN to predict thrombo-embolic stroke. The back propagation method was used as a prediction method and the accuracy achieved by this model was 89%. This paper addresses mobile health (m-Health) applications, IoT architecture, tools and technologies used in designing IoT systems, and a comparative analysis of various healthcare sensors and their classifications (Bhatia et al., 2020). The authors used deep convolutional neural networks for medical picture segmentation and examined well-known medical picture datasets, various criteria used to evaluate segmentation tasks and the efficacy of several CNN-based networks (Malhotra et al., 2022). IoT has a significant impact and is expanding in this industry as intelligence spreads to everything, including homes, cars, cities, farms, and other structures. Concerning the present widely used applications and the most recent research trends in this field, these papers highlight and contribute to the numerous facets of the Internet of Things (Sharma et al., 2021). In order to find the appropriate hyperparameter, this research optimized hyperparameters using Random Search, DL and Bayesian optimization. Scaling, smoothing, grayscale, morphological, thresholding operations were used to process the CT scan pictures. The authors described a study in which the Gray Level Co-occurrence Matrix (GLCM) extract features from pictures and feature selection was employed to select relevant features for classification. This study also used deep learning (DL) with hyperparameter settings for data classification and two optimization methods, namely, Bayesian Optimization and Random Search, were compared. The results proved that random forest performs best in terms of accuracy whereas Bayesian optimization has a smaller optimization time (Badriyah et al., 2019). The author proposed a technique that classified MR images of the brain into two categories, namely, normal and abnormal, where the abnormal category is further classified into various types of tumors. The proposed method used two classifiers MLP and C4.5 decision tree where maximum precision was 95% which was obtained using the MLP algorithm that considered 174 images (George et al., 2015).

Another study presented a novel CAD-BSDC model that classified MR images into two categories, named normal and abnormal classes. This technique has multiple processes like feature extraction, hyperparameter tuning, etc. A data set of T2-weighted MR brain images was used for experimental analysis and demonstrated good efficiency as compared to state of art techniques in terms of different performance metrics (Eshmawi et al., 2022). The authors explored ML algorithms for brain stroke classification using microwave imaging system and proposed a technique using distorted Born approximation for creating training data set on the basis of dielectric contrast space. Three algorithms, namely, SVM, MLP and k-NN were used where MLP showed the best results to identify presence or absence of stroke (Mariano et al., 2022). The authors explored the latest advancements in the domain of stroke and conducted a survey where 113 research papers were analyzed that were taken from different databases. They found some technical and non-technical challenges in the domain of stroke and also highlighted their future expectations. This work helps medical researchers to propose innovative techniques for the detection and segmentation of stroke (Karthik et al., 2020). A study classified CT images of the brain using a fully automatic system via DL. Mask R-CNN and transfer learning process is combined with ML techniques for the segmentation of stroke (Xu et al., 2020). The authors developed an automatic system using the DL algorithm for early prediction of stroke. This study used 256 images and achieve an accuracy of 90%. The authors used the CNN technique that helps medical researchers to diagnose stroke (Chin et al., 2017b). This research classified ML techniques into 4 categories on the basis of their functionalities. A literature review of 39 papers from 2007 to 2019 was conducted and 10 papers showed SVM as an optimal model for prediction of stroke. Also, CT images were used in the data set and the random forest was also chosen as an efficient technique (Sirsat M. S. et al., 2020). This study presented a technique for classifying into stroke and non-stroke categories of MRI images. They used the Gabor filter to extract characteristics which were then categorized using the MLP technique. The different number of features was considered to evaluate the results of the proposed method (Devi and Rajagopalan, 2013). This work uses supervised machine learning to provide a method for predicting blood cancer. To deal with imbalanced and high-dimensional data, it uses Chi-squared feature selection and ADASYN resampling. Logistic regression, support vector, and additional tree classifiers are all combined into one hybrid classifier called LVTrees. The suggested methodology outperforms state-of-the-art approaches in terms of accuracy, which has been verified by numerous experiments and statistical analyses (Rupapara et al., 2022). This study focuses on transfer learning-based pneumonia detection using X-ray pictures. The dataset is preprocessed in order to accommodate several pre-trained CNN models, including VGG16, Inception-v3, and ResNet50. These models are combined into ensembles, and Cohen’s kappa and AUC are used to assess performance. The best accuracy and recall are achieved by Inception-V3 with CNN (Mujahid et al., 2022). The strategy consists of two modules: Using disease symptoms, safety measures, and the associated dataset, Module 1 trains machine learning models using SVM, random forest, and other methods. Users of Module-2 can speak symptoms into a microphone to have them converted to text by Google Speech Recognition (Rustam et al., 2022). Deep learning can extract subtleties from huge datasets, so, the authors presented a neural network approach for trustworthy NSC fate forecasting. This approach correctly classifies cell types from unlabeled bright field photos, even after just 1 day of culture. With inducers including neurotrophins, hormones, chemicals, and nanoparticles, it exhibits great precision and robustness in a variety of test conditions, displaying its wide applicability (Zhu et al., 2021). This article proposes a technique for medical event extraction and focuses on specific characteristics of tumor-related medical events. The main tumor site, size features, and metastatic sites are all captured by the suggested standard extraction technique. A key-based strategy and a pseudo-data-generation technique are recommended due to the dearth of annotated texts in order to improve transfer learning across diverse tumor-related medical event types. Numerous tests on the CCKS2020 dataset confirm its effectiveness (Dhiman et al., 2022). According to this study, label-free and real-time depiction of diverse histologic characteristics in fresh prostate samples is possible using stimulated Raman scattering (SRS) microscopy. A diagnostic CNN that was trained on the photos of 61 patients can classify prostate cancer Gleason patterns with an accuracy of 85.7%. 22 cases of independent testing demonstrate an accuracy of 84.4%. In 21 cases, SRS-assisted deep learning generates Gleason ratings that are 71% consistent with the pathologists’ grading (Ao et al., 2023). This study makes the case that the challenging Edge environment demands specialized designs with autonomous computing. A key-value Edge data storage is first introduced in a prototype, which autonomously maintains latency for feature vectors while sacrificing accuracy for key frames. Early findings show a median latency improvement of 84.8% compared to non-autonomous operation, especially for dynamic scenarios and sporadic wireless interference (Ravindran and George, 2018). The goal of this study was to investigate the effects of ART treatment on NSPC proliferation and neurogenesis in a mouse model of MCAO. ART reduced the ischemic brain volume, according to an MRI. Immunofluorescence, electron microscopy, and diffusion tensor imaging all showed improvement in the white matter lesions brought on by ischemia. Following ischemia/reperfusion, endogenous NSPCs were activated by ART. Overexpression of FOXO3a counteracted the neuro-restorative effects of ART (Zhang et al., 2022). The main research gap identified was to check the impact of various optimizers on the accuracy of brain stroke detection using MLP. So, the authors tried to mitigate these gaps by proposed work. Table 1 is a tabular representation of existing literature in this domain.

**TABLE 1:** Comparison chart representing existing literature in this domain.

References	Year	Method used	Dataset used	Clinical implications
Singh et al. (2020)	2020	Comparative Analysis	Not specified	Analysis of various stroke prediction techniques. From the different methods applied, the composite method of DT, PCA and ANN gives the optimal result.
Nwosu et al. (2019)	2019	EHR Data Analysis	Electronic Health Records	A systematic analysis of risk factors for stroke prediction was done and DT, RF and NN were used for predicting stroke. As a result, the MLP model has the best performance with an accuracy of 75.02%.
Almadani et al. (2018)	2018	Data Mining Classification	Dataset obtained from Ministry of National Guards Health Affairs hospitals, Kingdom of Saudi Arabia	Prediction of stroke using data mining classification techniques and a data mining model was built with 95% accuracy.
Kansadub et al. (2015)	2015	Stroke Risk Prediction	Demographic data collected from the Faculty of Physical Therapy, Mahidol University, Thailand	Development of a stroke risk prediction model based on demographic data using DT, NB and NN models. DT is 75% accurate and NB has 0.769 AUC.
Khosla et al. (2010)	2010	Integrated Machine Learning	Cardiovascular Health Study (CHS) dataset	An integrated machine learning approach to stroke prediction.
Shanthi et al. (2009)	2009	Artificial Neural Network Model	Collected data from 50 patients	Designed an ANN model for the prediction of thrombo-embolic stroke with 89% accuracy.
Badriyah et al. (2019)	2019	Hyperparameter Optimized Deep Learning	Dataset having patient’s CT brain scan image	Improved stroke diagnosis accuracy using hyperparameter optimized deep learning. Random Search had the best accuracy, while Bayesian Optimization excelled in optimization time.
Eshmawi et al. (2022)	2022	Deep Learning Ensemble	Dataset which comprises T2-weighted MR brain images	An ensemble of deep learning-enabled brain stroke classification models using MRI images.
Mariano et al. (2022)	2022	Machine Learning Algorithms	Dataset created via microwave imaging systems	Brain stroke classification via ML algorithms (SVM, MLP, k-NN) trained with a linearized scattering operator.
Karthik et al. (2020)	2020	Neuroimaging and Deep Learning	Not used	Review of 113 research papers on neuroimaging and deep learning advancements in brain stroke detection.
Xu et al. (2020)	2020	Deep Learning for Brain CT Scans	356 images of Brain CT Scans	Deep learning analysis of brain CT scans for hemorrhagic stroke patients.
Chin et al. (2017b)	2017	CNN Deep Learning Algorithm	CT images of Ischemic Stroke Data	Automated early ischemic stroke detection using a CNN deep learning algorithm.
Sirsat et al. (2020b)	2020	Machine Learning Review	Not used	A review of machine learning applications on various datasets for brain stroke detection.
Devi and Rajagopalan (2013)	2013	Multi-Layer Perceptron with Watershed Segmentation and Gabor Filter	52 DWI scan images	Brain stroke classification based on an MLP using watershed segmentation and Gabor filter.

A thorough literature research was carried out to ensure comprehensive analysis using the Scopus database. The papers of time duration used were from January 2018 to May 2023 for the most relevant search in this field. The keywords used for the specific search were [“brain stroke” AND (“machine learning” OR “deep learning”)]. 108 total papers were identified which were selected based on their relevance to align with the objectives of the proposed study. The papers from various sources like research articles, review papers and conference papers were considered.

**FIGURE 3:**
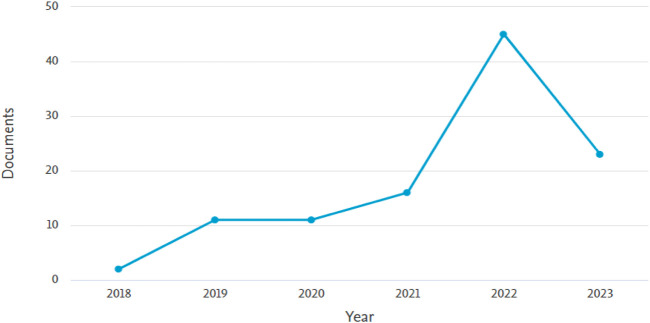
Trends in the number of articles published each year related to brain stroke research.

Figure 3 clearly illustrates a substantial and rapid increase in the number of papers related to brain stroke research from 2018 to 2022. This trend indicates a growing interest in the field among researchers. The number of publications experienced significant growth from 2018 to 2021 and reached its peak in 2022, with a remarkable count of 45 publications. While the data for 2023 is not yet complete, it is anticipated that there will be a moderate trend compared to the previous year. By observing the increasing trend in publications, it becomes evident that the scientific community is actively engaged in exploring innovative approaches, technologies, and interventions for brain stroke. The heightened interest in this field may lead to advancements in prevention, diagnosis, treatment, and overall management of brain stroke cases.

**FIGURE 4:**
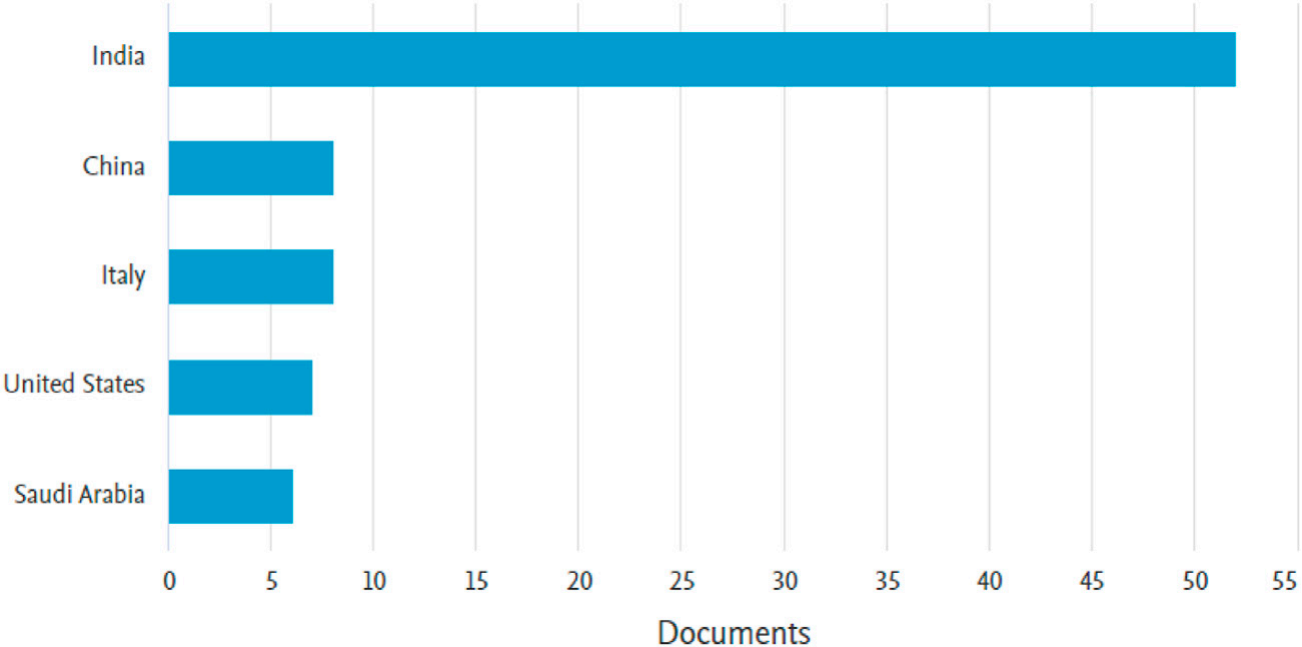
Visual representation of top five countries with highest number of publications in field of brain stroke.

Regarding the geographical distribution, a total of 108 documents were published, originating from 35 different countries and regions. The country with the highest number of published papers on brain stroke is India, contributing 52 papers (48.14% of the total). China follows closely with 9 papers (8.3%), and Italy ranks third with 8 papers (7.4%). These findings highlight the significant contributions made by these three countries in the field of brain stroke research. Figure 4 showcases the top five countries/regions contributing to the research in this field.

**FIGURE 5:**
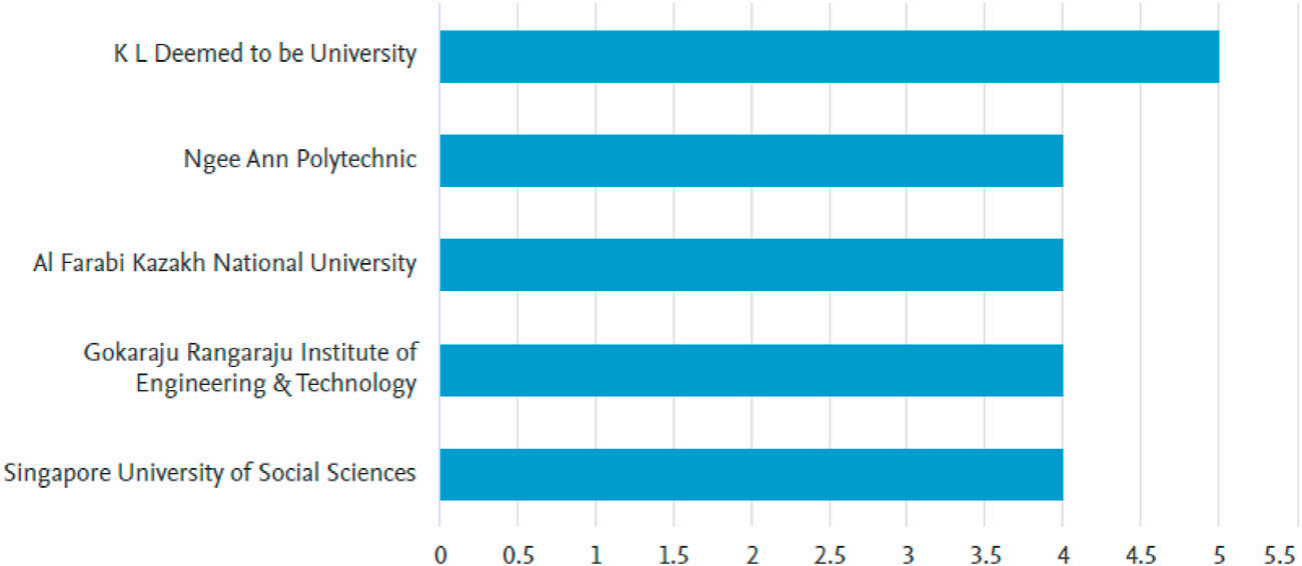
Data of top five institutions that worked related to brain stroke.

Figure 5 presents the ranking of the top five institutions based on their productivity in terms of publications on brain stroke. The institution with the highest productivity is the K L Deemed to be University, contributing 5 publications (4.62% of the total). Following closely, Ngee Ann Polytechnic and Al Farabi Kazakh National University both recorded 4 publications each (3.7% each). These institutions have demonstrated their significant contributions to the field of brain stroke research.

## 3 Materials and methods

### 3.1 Dataset

Using deep learning to predict the probability of a brain stroke is an interesting and important application of machine learning. Kaggle is a popular platform for finding and working with datasets, so a dataset is chosen from here only. The dataset has numerous physiological attributes as its characteristics (Stroke Prediction Dataset, 2021). This can allow the DL model to learn complex relationships between the characteristics and the likelihood of a stroke. However, the quality and completeness of the dataset are crucial to the success of the model. It is also important to properly preprocess and clean the data to ensure that the model is not learning from irrelevant or noisy information. However, using deep learning for medical applications such as stroke prediction can have a significant impact on patient outcomes and healthcare in general. A Kaggle dataset was utilized for stroke prediction. There are 4,981 rows and 11 columns present in the dataset. The primary attributes are “gender”, “age”, “heart disease”, “hypertension”, “ever married”, “residence type”, “work type”, “avg glucose level”, “smoking status”, “bmi” and “stroke.” The output field “stroke” has either value “1” or “0”. “0” implies no risk of stroke while “1” indicates a stroke risk. It is common to encounter imbalanced datasets in machine learning, where one class is much more prevalent than the other. In the case of stroke prediction, a value of “0” (indicating no stroke) would be more common than a value of “1” (indicating a stroke), since strokes are relatively rare events. So, pre-processing of data is done to balance data to get improved accuracy (Islam et al., 2021).

### 3.2 Deep Learning

Deep learning is a sub-field of ML that uses artificial neural networks to carry out complex computations on extensive datasets. Deep learning algorithms have the ability to process diverse types of data and tackle complex problems, necessitating substantial computational resources and extensive information. A class of feed forward ANN is called a multilayer perceptron which is the most fundamental deep neural network which consists of several fully linked layers. Each layer is composed of many nonlinear functions which display the weighted sum of all outputs from previous layers. A multi-layer perceptron has an input layer for every input, an output layer for every output having one neuron each in input as well as output layer, and any count of hidden layers with any count of neurons on every hidden layer. A non-linear function, namely, rectified linear activation (ReLU) outputs the input directly if input is +ve but it outputs 0 if no inputs. ReLU activation functions are utilized by each node in multi-layer perception. The first two dense layers—which are also hidden layers—are used to create a fully connected model. The output layer, the final densest layer, has 20 neurons that select the category to which the data belongs. Here, a compile function that uses metrics, optimizers and loss is implemented.

### 3.3 Deep Learning optimizers

In the field of deep learning, optimizers play a vital position in adjusting parameters of a model. The primary objective of an optimizer is to optimize model weights in order to maximize the performance of a specified loss function. The loss function serves as a metric for evaluating the efficacy of the model. Consequently, during the training process of a neural network model, it is essential to use an optimizer to iteratively adjust the weights and enhance the model’s performance. Cross-entropy loss or log loss measures how well the model performs where output of classification model is probability value between 0 and 1. If the expected value does not match the actual value, it rises (Islam et al., 2022; Aggarwal et al., 2023). Neural network model uses optimizers, which are algorithms, to update each layer’s weights and learning rates after each iteration in order to minimize losses. The Adam version of gradient descent has an enhancement called AdaMax (Wang et al., 2022) that expands on the concept of the infinite norm (max) and might lead to more successful optimization as shown in Eqs 1–4 below.

$$w_{t+1}=w_{t}-\frac{\alpha }{v_{t}}∙\hat{m}t$$ (1)

where

$$\hat{m}t=\frac{mt}{1-\beta t1}$$ (2)

is the bias correction for m and

$$m_{t}=\beta_{1}m_{t-1}+\left(1-\beta_{1}\right)\frac{dL}{dw_{t}}$$ (3)

$$v_{t}=\max\left(\beta_{2}v_{t-1},\left|\frac{dL}{dw_{t}}\right|\right)$$ (4)

with m and v initialized to 0.

RMSProp is an optimization algorithm that is an extension of gradient descent and is similar to AdaGrad in that it also measures the size of the step for every parameter. However, instead of using the cumulative sum of gradients squared, RMSProp uses a decaying average of the squared gradients as shown in Eqs 5–6 below.

$$w_{t+1}=w_{t}-\frac{\alpha }{\sqrt{v_{t}+\epsilon }}\;\frac{dL}{dw_{t}}$$ (5)

where

$$v_{t}=\beta v_{t-1}+\left(1-\beta \right)\left[\frac{dL}{dw_{t}}\right]2$$ (6)

and v initialized to 0.

This allows the algorithm to adapt to changing conditions in the optimization problem and converge faster. Adadelta is a gradient descent addition that relies on AdaGrad and RMSProp (Stroke Prediction Dataset, 2021). It modifies the calculation of the custom step size to ensure that the units are consistent, which eliminates the need for an initial learning rate hyperparameter as shown in Eqs 7–10 below.

$$w_{t+1}=w_{t}-\frac{\sqrt{D_{t-1}+\varepsilon }}{\sqrt{v_{t}+\epsilon }}∙\frac{dL}{dw_{t}}$$ (7)

where

$$D_{t}=\beta D_{t-1}+\left(1-\beta \right)\left[∆w_{t}\right]2$$ (8)

$$v_{t}=\beta v_{t-1}+\left(1-\beta \right)\left[\frac{dL}{dw_{t}}\right]2$$ (9)

with D and v initialized to 0, and

$$∆w_{t}=w_{t}-w_{t-1}$$ (10)

CrossEntropyLoss (Feng et al., 2023) is the loss function and AdaMax, RMSProp and Adadelta are the optimizers used in the proposed model. Epochs indicate how many forward and backward passes the model will undergo training; in the proposed model, it is 200. The batch size indicates the quantity of samples which is 32. A validation split is a float value that lies between 0 and 1. In the proposed model, it is 0.5. This portion of the training data will be kept aside by the model to be used after each epoch to assess the loss and any model metrics. In the suggested model, it is 0.5.

### 3.4 Proposed methodology

Various datasets were carried out with respect to moving with the implementation. Once the dataset was chosen, the work moved on to data preprocessing, which involves cleaning, transforming and preparing the data for analysis.

**FIGURE 6:**
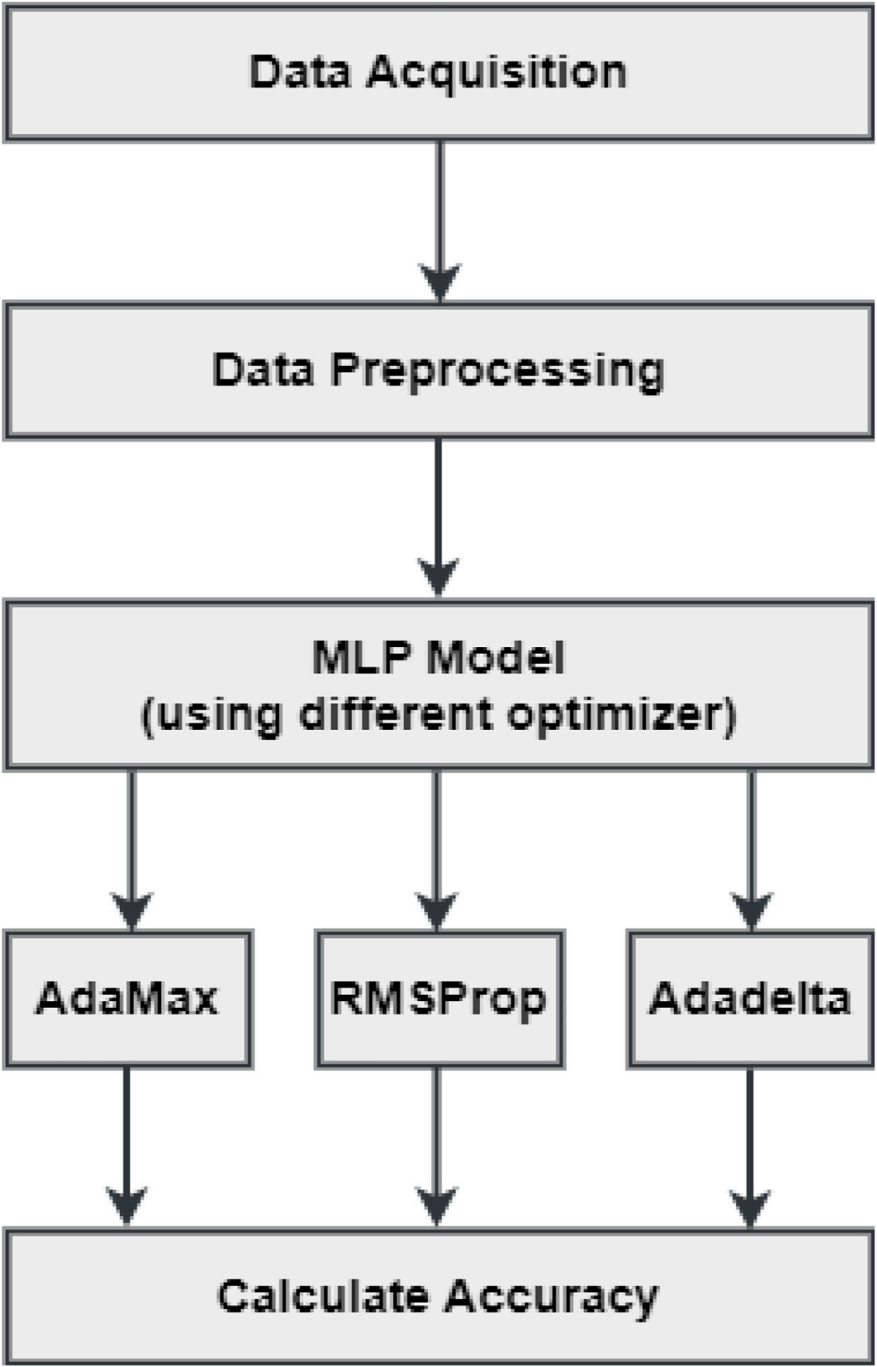
Flowchart of the proposed model.

The data preprocessing stage included handling missing values, balancing the data (if it was imbalanced) and performing label encoding. Handling missing values involves dealing with any cells in the dataset that do not have a value or have a "null" value. This can be done by imputing the missing values or extracting the columns or rows with missing values, relying on the context of the research. Balancing the data is necessary when the dataset contains an unequal count of instances for individual classes that can impact the performance of model. The preprocessed data is now ready for model construction. MLP technique is applied to the preprocessed dataset for model construction. The best optimizer is determined by a comparison of the model’s accuracy. The flow chart of the methodology of proposed system is depicted in Figure 6.

## 4 Experimentation and analysis

In this section, the results of the proposed work are presented, where the dataset is first cleaned in order to comprehend the deep learning model. Then, it is examined for null values during data preprocessing and gaps are filled suitably. After that, the dataset is split into a train as well as a test data so that it can be preprocessed. The model is trained x_train and y_train using the fit technique, totaling 200 epochs. Additionally, the dataset is split into two sets: one with validation of 0.1, which means that 90% of training data train model and rest 10% validate it, and another with validation of 0.2, which means 80% of training data train model and rest 20% of the training data validate it. The most accurate prediction model is then found by comparing models that were created using the new data and a classification method to calculate accuracy. After thorough analysis, the research concludes which algorithm is best for the prediction of stroke. The objective of the work is to develop a model which can accurately predict brain stroke. The proposed model is tested using various optimizers and the results varied in accuracy.

**FIGURE 7:**
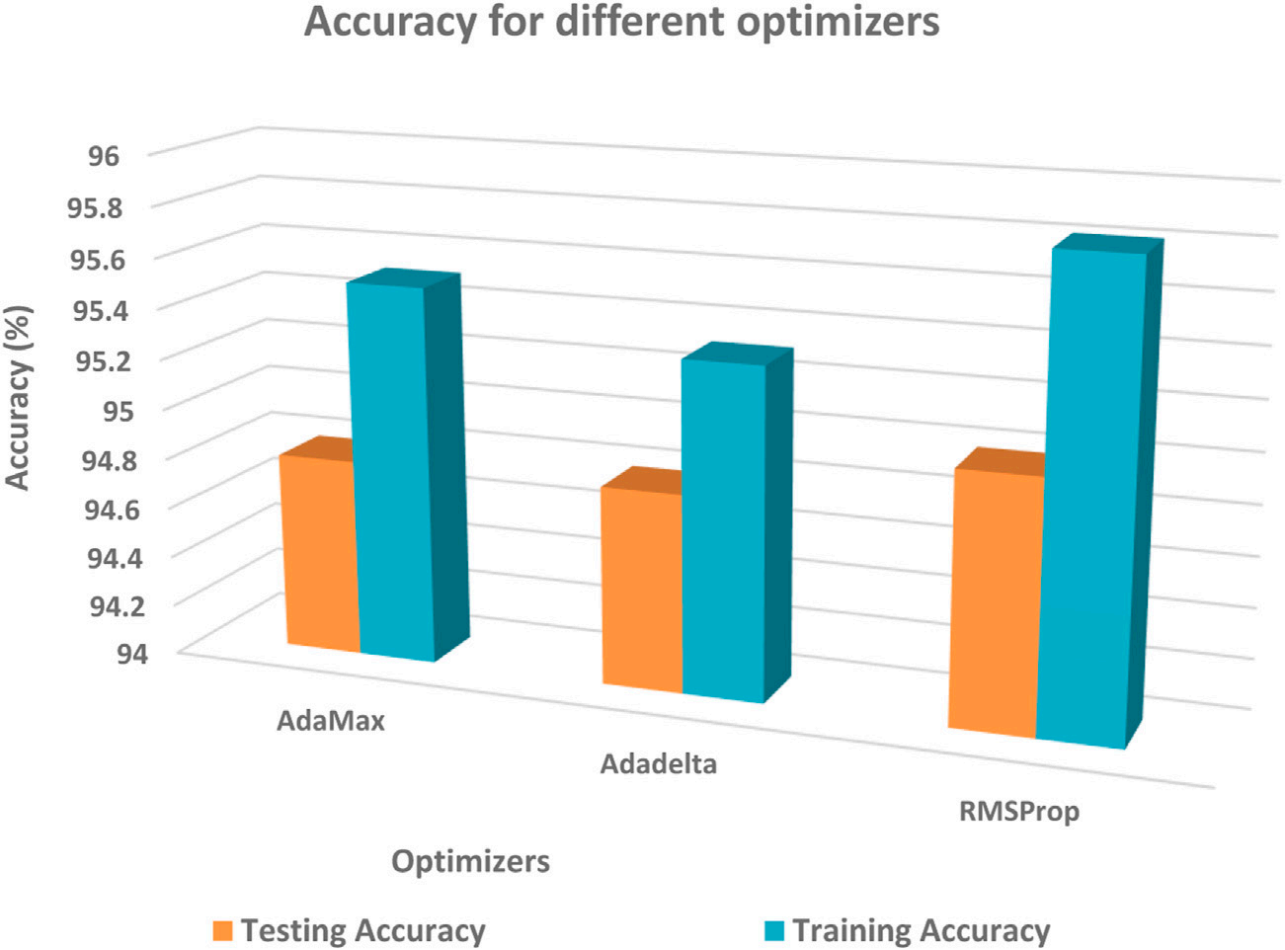
Accuracy on test dataset with an 80–20 split.

**FIGURE 8:**
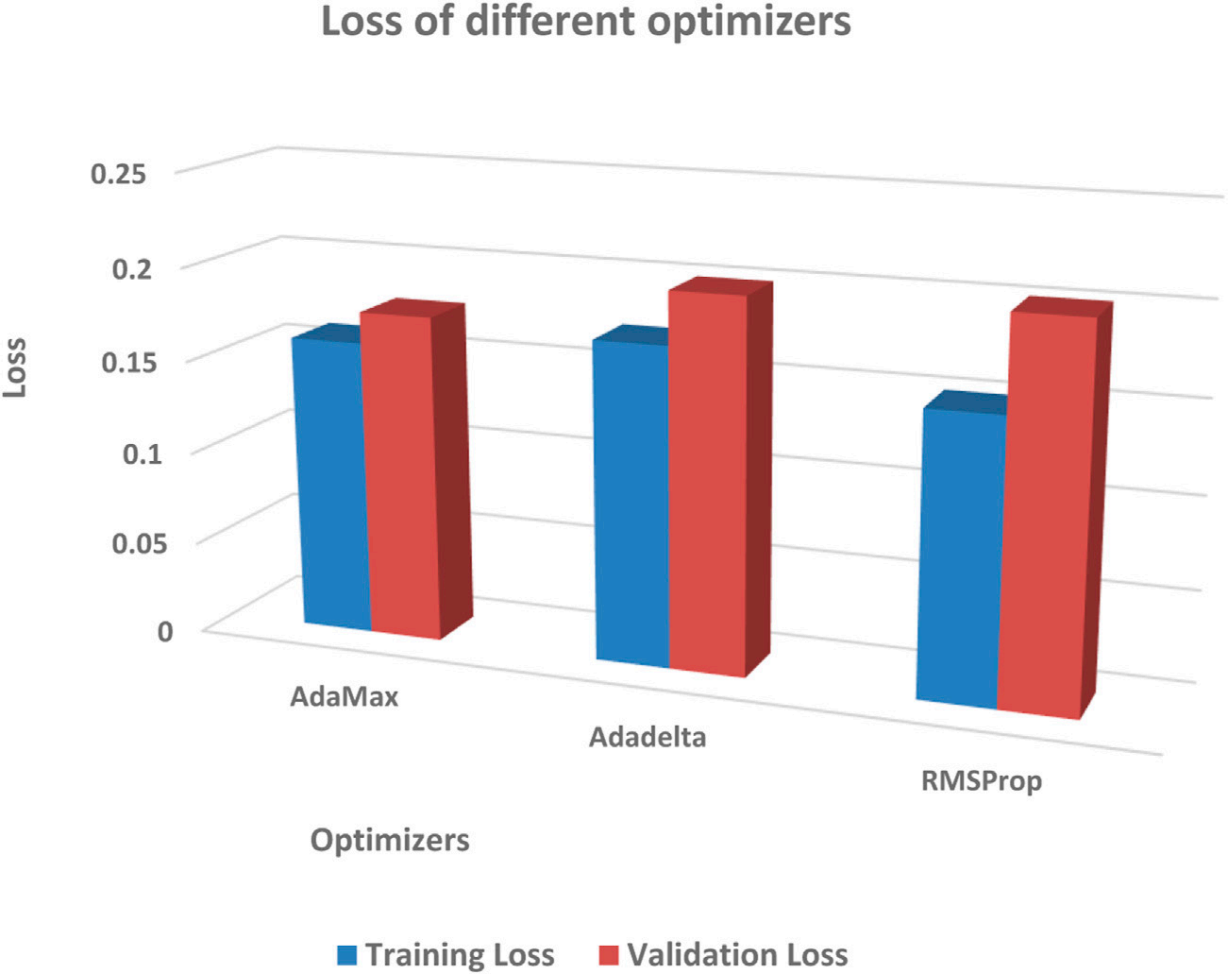
Loss on train set for 80–20 split.

The ratio of training to testing data is maintained at 80% for the training data and 20% for the testing data. As demonstrated in Figure 7; Figure 8, the RMSProp optimizer for this particular ratio produced an accuracy of 94.98% and a loss of 0.15 at 0.01 learning rate.

Testing and training data are separated from dataset for maintaining the 90% training and 10% testing data ratio that will result in greater accuracy and efficiency for this task.

**FIGURE 9:**
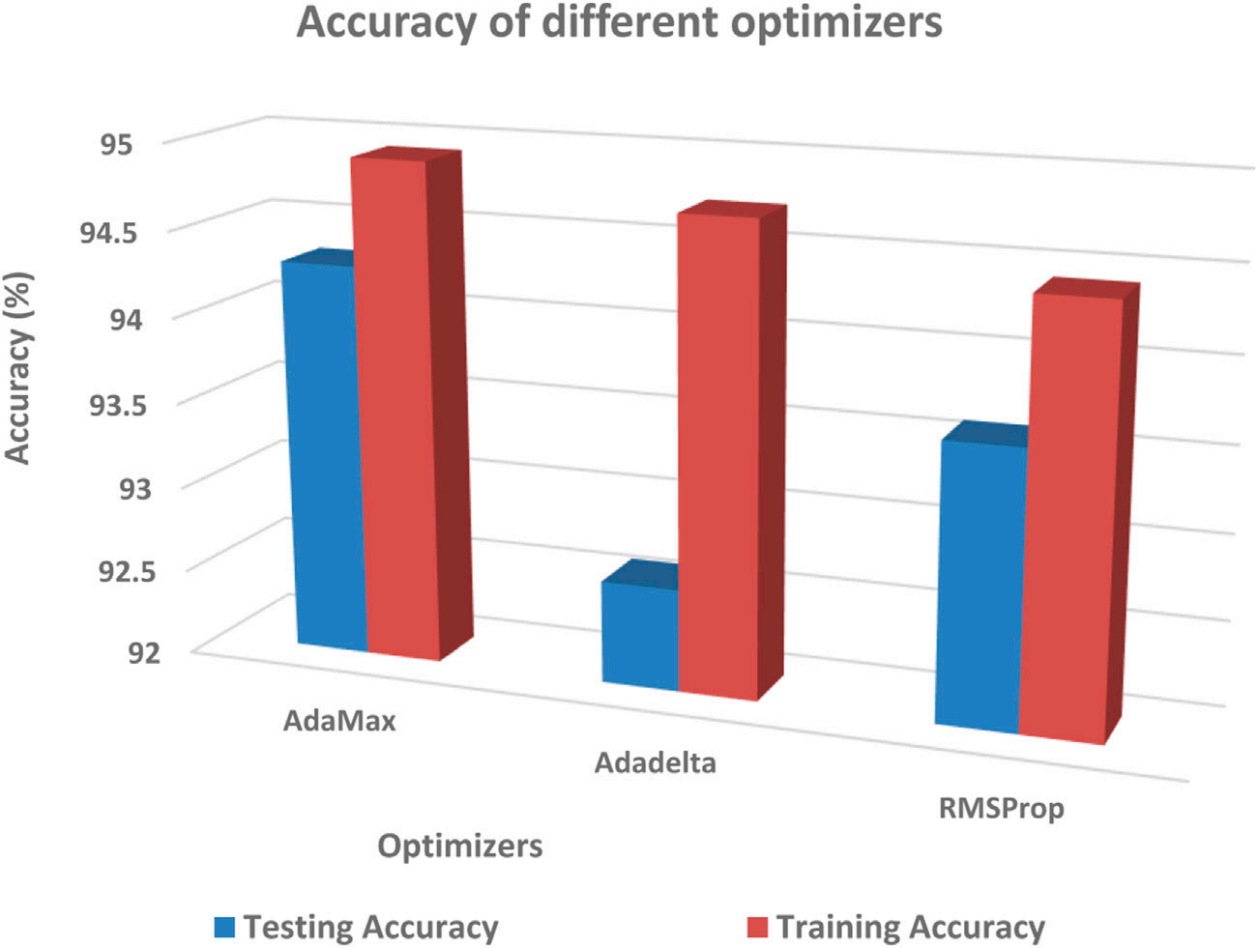
Accuracy on test set with a 90–10 split.

**FIGURE 10:**
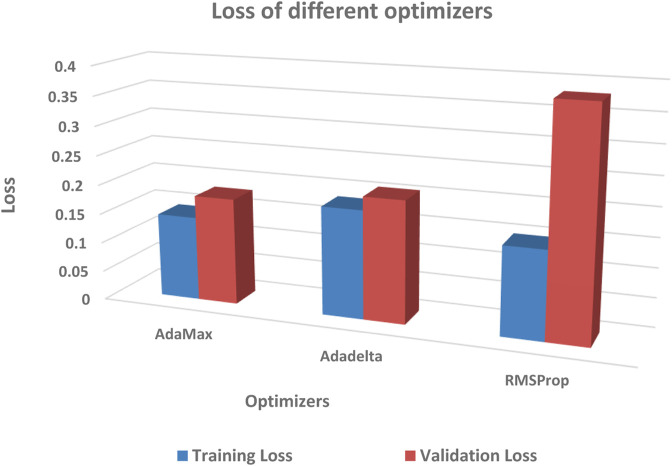
Loss on train set for 90–10 split.

According to Figures 9; 10, the AdaMAx optimizer achieved an accuracy of 94.3% and a loss of 0.14 for this particular ratio at 0.01 learning rate.

**FIGURE 11:**
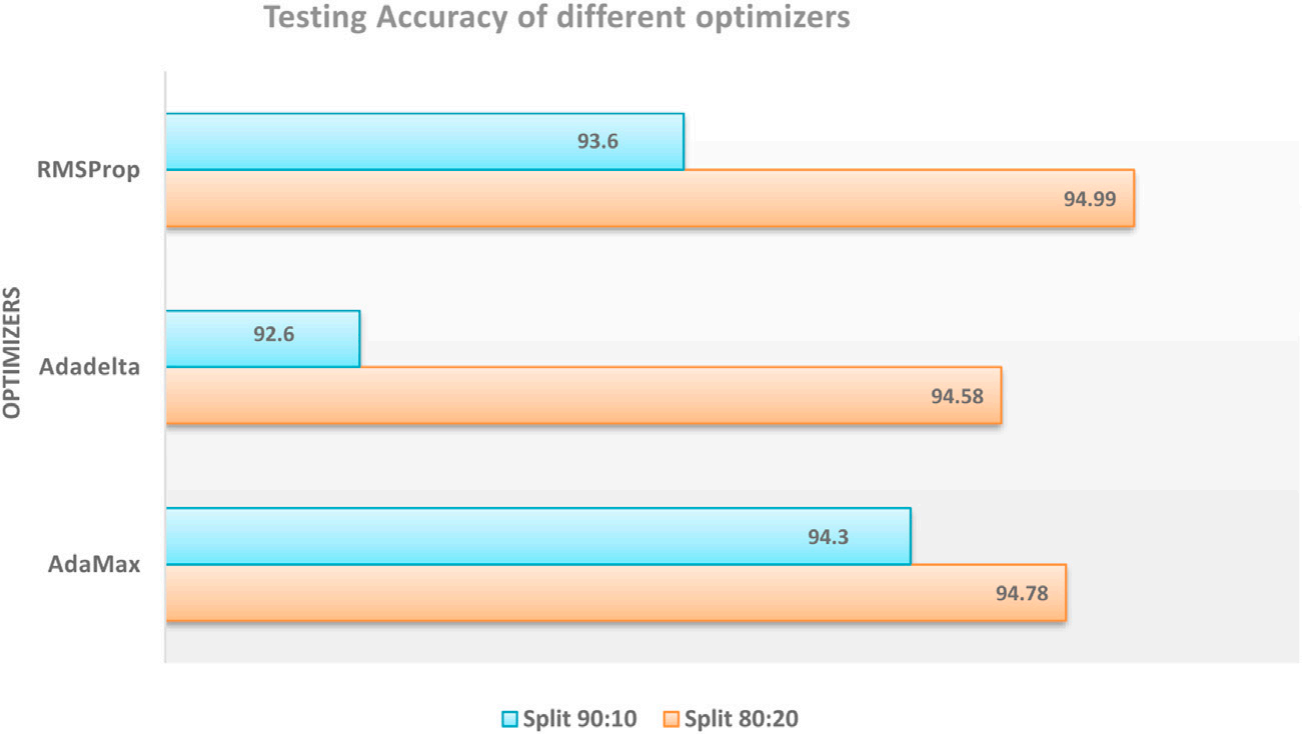
Comparison of testing accuracy across multiple optimizers.

**FIGURE 12:**
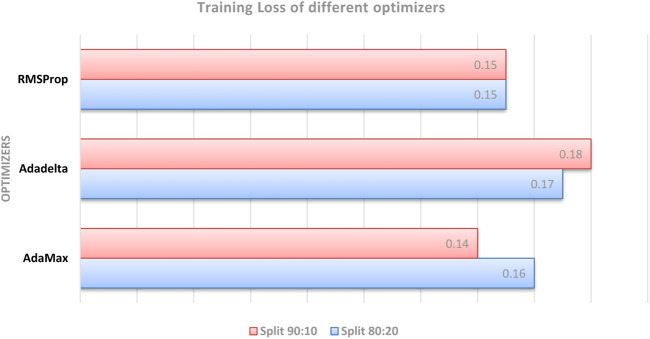
Comparison of training loss across multiple optimizers.

The performance of different optimizers was evaluated by calculating accuracy and loss on the dataset. Figure 11; Figure 12 illustrate the variations in accuracy and loss achieved using different optimizers. These metrics provide insights into the effectiveness of each optimizer in terms of the model’s predictive capabilities and the level of dissimilarity between predicted and actual values. By analyzing these figures, it is possible to compare the performance of different optimizers and identify the ones that yield the best results. This information can be valuable for selecting the most suitable optimizer for enhancing the accuracy and minimizing the loss in the model.

Among the tested optimizers, the RMSProp optimizer demonstrated the highest performance in terms of testing accuracy. It achieved an accuracy of 94.78% in the dataset split of 80:20. This result indicates that the RMSProp optimizer was effective in improving the model’s accuracy in predicting the desired outcome. The findings highlight the potential of the RMSProp optimizer for optimizing the model’s performance in similar tasks or datasets.

During the experimentation, the AdaMax optimizer exhibited notable performance in terms of training loss. Specifically, on the dataset split of 80:20, it yielded a training loss of 0.16. In the 90:10 dataset split, the AdaMax optimizer achieved an even lower training loss of 0.14. These results indicate that the AdaMax optimizer effectively minimized the discrepancy between the predicted and actual values during the training process. The lower training loss suggests that the model trained using the AdaMax optimizer was able to learn the underlying patterns and relationships in the data more accurately. Consequently, this can contribute to enhanced predictive capabilities and more reliable outcomes. The findings emphasize the potential of the AdaMax optimizer for optimizing the training phase and improving the overall performance of the model.

This study concentrated on developing a model for accurately predicting brain strokes. The dataset was splited into 2 set, namely, training and testing data with different ratios used for validation. Various optimizers were evaluated to determine their impact on accuracy and loss. The RMSProp optimizer demonstrated the highest testing accuracy of 94.78% in the 80:20 dataset split, while the AdaMax optimizer achieved the lowest training loss of 0.14 in the 90:10 dataset split. These findings highlight the effectiveness of these optimizers in improving the model’s performance. The study concludes that optimizer selection plays a crucial role in stroke prediction accuracy. This research can contribute to the development of more reliable models for the early detection and treatment of strokes.

## 5 Conclusion and future scope

A stroke is a dangerous medical condition that requires prompt treatment to prevent worsening. The development of a deep learning model can assist in the early diagnosis of stroke and decrease future severe effects. The practical consequences of improved brain stroke detection accuracy are significant. Quick and accurate stroke detection improves actions, treatment approaches, and misdiagnosis. This improves patient outcomes, disability, and death. More efficient healthcare resource allocation reduces needless operations and improves patient management. Accurate detection increase telemedicine and stroke care research and innovation. These accuracy gains promote patient-centered treatment, healthcare system efficiency and society. The aim of the proposed model is to raise the standard of stroke diagnosis, enabling doctors to identify stroke quickly and reliably and to begin the appropriate treatment as soon as feasible. This study investigates the performance of the MLP algorithm with different optimizers in predicting stroke based on various physiological variables. The RMSProp optimizer, which was selected, outperforms the other two with an accuracy of 94.98% on an 80–20 split and a learning rate of 0.01. Moreover, the AdaMax optimizer produces a 90–10 split with a learning rate of 0.01 with the least loss of 0.14. By taking into account multiple optimizers and various learning rates, performance comparisons may be made. The data used in this work is solely textual, but gathering a dataset of CT scans to forecast the chance of stroke can be more effective in the upcoming future. Also, the strengths of diverse ML and DL techniques can be combined to construct an ensemble model that could lead to improved robustness and better predictive capabilities.

## Data Availability

The original contributions presented in the study are included in the article/Supplementary Material, further inquiries can be directed to the corresponding authors.
